# How to Differentiate Sites of Gastrointestinal Bleeding in Patients with Hematochezia by Using Clinical Factors?

**DOI:** 10.1155/2013/265076

**Published:** 2013-11-18

**Authors:** Yuwares Sittichanbuncha, Suthasinee Senasu, Theerayut Thongkrau, Chaiyapon Keeratikasikorn, Kittisak Sawanyawisuth

**Affiliations:** ^1^Emergency Medicine Department, Faculty of Medicine, Ramathibodi Hospital, Mahidol University, Bangkok 10400, Thailand; ^2^Department of Computer Sciences, Faculty of Sciences, Khon Kaen University, Khon Kaen 40002, Thailand; ^3^Department of Medicine, Faculty of Medicine, Khon Kaen University, Khon Kaen 40002, Thailand; ^4^Research and Training Center for Enhancing Quality of Life of Working-Age People, Khon Kaen University, Khon Kaen 40002, Thailand

## Abstract

Hematochezia is one of common gastrointestinal complaint at the Emergency Department (ED). Causes may be due to upper (UGIB) or lower (LGIB) gastrointestinal tract bleeding. Here, clinical factors were studied to differentiate sites of bleeding in patients with hematochezia. All patients with an age of more than 18 years who were diagnosed with GIB at the ED, Ramathibodi Hospital, Thailand were enrolled. Patients who presented with hematochezia and received complete workups to identify causes of bleeding were studied and categorized as being in the UGIB or LGIB groups. There were 1,854 patients who presented with GIB at the ED. Of those, 76 patients presented with hematochezia; 30 patients were in the UGIB group, while 43 patients were in the LGIB group. Clinical variables between both groups were mostly comparable. Three clinical factors were significantly associated with UGIB causes in patients with hematochezia including systolic blood pressure, hematocrit level, and BUN/Cr ratio. The adjusted odds ratios for all three factors were 0.725 (per 5 mmHg increase), 0.751 (per 3% increase), and 1.11 (per unit increase). Physicians at the ED could use these clinical factors as a guide for further investigation in patients who presented with hematochezia.

## 1. Introduction

Gastrointestinal bleeding (GIB) is a common complaint at the Emergency Department (ED). GIB is classified as upper (UGIB) or lower (LGIB) anatomically at the ligament of TREIZ. Further investigations or management strategies are different between UGIB and LGIB patients. Gastroscopy is indicated in UGIB patients to identify causes of UGIB, while colonoscopy and/or vascular scans are investigations of choice for LGIB.

An obvious symptom of UGIB is hematemesis or having melena. Not all of patients with UGIB, however, present with hematemesis or melena [1]. Hematochezia is a presenting symptom of LGIB. Patients with UGIB may also present with hematochezia. Nasogastric lavage may not be a useful procedure if the patients do not have hematemesis [2]. Clinical factors such as the history of alcohol consumption, smoking, or NSAID use were found to be related to UGIB in previous studies [3]. Here, clinical factors were studied to determine if they could differentiate sites of GIB presenting with hematochezia at the ED.

## 2. Methods

All patients with an age of more than 18 years who were diagnosed with GIB at the ED, Ramathibodi Hospital, Mahidol Universtiy, Bangkok, Thailand, were enrolled. The study period was between December 2003 and December 2007. Patients were included if they presented with hematochezia and received further investigation to identify causes of GIB. Patients who had bleeding from internal hemorrhoids, anal fissures, or local causes at the anus were excluded.

Clinical factors were reviewed and extracted from medical records. These factors were age, gender, history of GIB, history of cancer, comorbid diseases such as cirrhosis, alcohol intake within 3 months, smoking within 3 months, medications, history of abdominal pain, vital signs, abdominal examination, characteristics of stool, blood tests for complete blood count, serum blood urea nitrogen (BUN), serum creatinine (Cr), serum albumin, and results of gastroscopy or colonoscopy. All laboratory results were values at presentation at the ED.

Patients were categorized as being in either the UGIB or the LGIB group by the results of gastroscopy and/or colonoscopy. Clinical factors between both groups were compared by descriptive statistics. Clinically important factors or significant factors by univariate logistic analyses were included in the multivariate logistic analyses to identify factors associated with sites of GIB. Analytical results were presented in terms of crude odds ratios (OR), adjusted OR, and their 95% confidence intervals (CI). To demonstrate the discriminatory power or accuracy of the model, *c* statistics or the area under the receiver operating characteristic (ROC) curve was tested. All analyses were computed by SPSS version 11.2.

## 3. Results

During the study period, there were 1,854 patients presented with GIB at the ED. Of those, 1,698 patients were excluded due to having either hematemesis (1,390 patients) or obvious anorectal etiology (215 patients) or no investigations to identify causes of GIB (93 patients). There were 206 patients on whom gastroscopy and/or colonoscopy was performed to identify causes of GIB. Of those, 155 patients were diagnosed as UGIB and 43 patients had LGIB. The other 8 patients had no identified causes of GIB. The most common causes of UGIB and LGIB were gastric ulcer/duodenal ulcer/gastritis and colonic polyps (Table 1). Thirty patients (19.35%) in the UGIB group presented with hematochezia, while all 43 patients with LGIB presented with hematochezia.

Most clinical variables between patients who presented with hematochezia from UGIB and LGIB were comparable. There were eight significantly different factors between these two groups (Tables 2 and 3). Patients in UGIB group had a higher proportion of patients with history of cirrhosis (*P* value 0.025), history of alcohol consumption (*P* value 0.001), history of epigastric pain (*P* value 0.009), lower systolic blood pressure (*P* value < 0.001), lower diastolic blood pressure (*P* value 0.025), lower hematocrit level (*P* value 0.001), a higher BUN level (*P* value 0.024), and a higher BUN/Cr ratio (*P* value < 0.001). The ranges of BUN levels in the UGIB and LGIB groups were 4–70 and 3–103 mg/dL. All patients in the UGIB group had nasogastric lavage and bleeding was found in 15 patients (50%), while 23 patients (53.49%) in the LGIB had nasogastric lavage and none of these patients had bleeding from the nasogastric tube.

**Figure 1:**
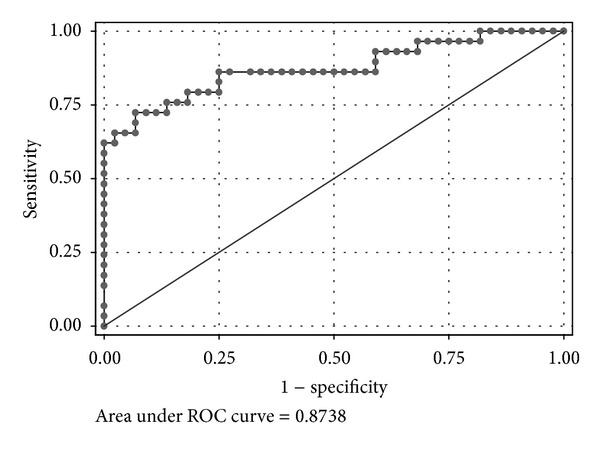
The receiver operating characteristic (ROC) curve of the predictive model for having upper gastrointestinal bleeding in patients presenting with hematochezia at the emergency department by multiple logistic regression analysis. The area under ROC curve was 0.874 (95% confidence interval of 0.783, 0.965) which indicates the accuracy of the model using systolic blood pressure, hematocrit level, and BUN/Cr ratio.

There were three factors significantly associated with UGIB in patients presenting with hematochezia by multivariate logistic analysis (Table 4). Systolic blood pressure and hematocrit levels were negatively correlated with UGIB. The adjusted odds ratios of both factors were 0.725 and 0.751. The adjusted odds ratio of the BUN/Cr ratio was 1.110 with a 95% confidence interval of 1.030 and 1.190. The ROC of the model is shown in Figure 1 with an area under the ROC of 0.874 (95% confidence interval 0.783 and 0.965).

## 4. Discussion

In resource-limited settings, proper and prompt investigation is needed to save costs of treatment and provide proper management. Choosing the appropriate investigation to identify causes of GIB in patients presenting with hematochezia in the ED is crucial. In this study, 19.35% of UGIB patients presented with hematochezia compared with 100% of patients with LGIB. The nasogastric lavage showed bleeding in 50% of UGIB patients and no bleeding in LGIB patients [4]. Nasogastric lavage is helpful to differentiate sites of bleeding in patients with only hematemesis [2].

Significant histories such as history of cirrhosis, alcoholic consumption, smoking, or epigastric pain were all suggestive of UGIB. These factors, however, were not strong enough to be independently associated with sites of bleeding in hematochezia patients. Only systolic blood pressure, hematocrit level, and the BUN/Cr ratio were more objective and could be used to differentiate sites of bleeding significantly and independently (Table 4).

All three of these factors may be associated with a large amount of UGIB [5]. UGIB tends to have a larger volume of bleeding than LGIB. The BUN/Cr ratio has been shown to be a factor to differentiate UGIB from LGIB [6–9] particularly if the ratio is more than 30 [10]. The median of the BUN/Cr ratio in this study was 29. The ratio also suggests the severity of UGIB [11]. The reason for having a high BUN/Cr ratio in UGIB is still controversial, possibly prerenal azotemia or high protein absorption from intestine.

In patients presenting with hematochezia, the BUN/Cr ratio is also a stronger factor to differentiate the site of GIB than the use of nasogastric aspiration [10]. Literature, however, recommends nasogastric aspiration to rule out UGIB in patients with hematochezia, particularly those with history of UGIB and a low hemoglobin level [12]. Other suggestive factors of UGIB in patients with hematochezia were a history of black stools and an age of less than 50 years [10].

Systolic blood pressure and hematocrit level are also independent factors to allocate the site of GIB in patients with hematochezia. The adjusted odds ratios for both factors were 0.725 per 5 mmHg of increasing systolic blood pressure and 0.751 per 3% of increasing hematocrit. In other words, a low systolic blood pressure and a low hematocrit level are suggestive of UGIB in patients with hematochezia. These results are similar to previous reports [10, 12]. The mean hematocrit level of patients in UGIB group in this study was 26.4%, while Witting et al. found that a hematocrit level of less than 30% was a significant predictor for UGIB in patients with hematochezia [10]. Those patients who have UGIB and a low systolic blood pressure of less than 100 mmHg, plus a hemoglobin less than 10 g/dL, and a hematocrit of less than 30%, or having hematochezia have a high risk for morbidity and rebleeding particularly in the elderly [13–16].

The rate of UGIB in patients with hematochezia has varied between 6.6–14% [12, 17]. In this study, the rate was quite high at 41.10%. The previous studies were conducted in hospitalized patients and the bleeding was occult and not active [12]. The patients in the current study were more severely ill and had active GIB at the emergency department. Byers et al. found that the most common cause of UGIB in 9 patients who presented with hematochezia was erosive gastritis in 4 patients or 36% [12]. This study found a similar finding with a larger study population. Fifteen out of 30 patients in the UGIB group had gastritis (50%) as shown in Table 1.

There are some limitations in this study. Retrospective data collection caused data loss or incomplete data. In addition, the formula provided needs to be confirmed elsewhere. However, the formula will be a useful tool for clinicians in resource-limited settings to choose the further appropriate investigations according to the site of GIB. Physicians should be aware that, in patients who present with hematochezia, UGIB is usually listed as the common possible cause [12, 17]. Prospective data collection to verify the model and cost saving regarding appropriate investigation and management strategies in patients with hematochezia are also needed.

## 5. Conclusion

Factors that may differentiate sites of bleeding in patients with hematochezia are systolic blood pressure, hematocrit level, and BUN/Cr ratio.

## Figures and Tables

**Table 1:** Causes of upper and lower gastrointestinal bleeding in patients presented with hematochezia.

Upper gastrointestinal bleeding *N* = 30	Lower gastrointestinal bleeding *N* = 43
Gastritis 15 (50.00%)	Colonic polyp 14 (32.55%)
Gastric ulcer 9 (30.00%)	Colitis 10 (23.26%)
Duodenal ulcer 2 (6.70%)	Tumor/malignancy 9 (20.93%)
Esophageal varice 2 (6.70%)	Diverticulitis 8 (18.6%)
Duodenitis 1 (3.30%)	Angioplasia 1 (2.30%)
Ulcerative mass 1 (3.30%)	Telangiectasia 1 (20.30%)

**Table 2:** Baseline characteristics of patients presented with hematochezia categorized by sites of bleeding as upper (UGIB) or lower (LGIB) gastrointestinal bleeding.

Variables	UGIB group N = 30	LGIB group *N* = 43	*P* value
Mean (standard deviation) age, year	60.43 (19.3)	62.19 (16.2)	0.675
Male gender	15 (50.0)	20 (46.5)	0.956
History of previous GI bleeding	10 (33.3)	5 (11.6)	0.050
Cancer	5 (16.7)	6 (14.0)	0.752
Cirrhosis	4 (13.3)	0	0.025
Alcohol consumption	9 (30.0)	1 (2.3)	0.001
Smoking	3 (10.0)	0	0.065
Medications			
None			
NSAIDs	17 (56.7)	34 (79.1)	0.093
Warfarin	10 (33.3)	6 (14.0)	0.509
Clopidogrel	0	2 (4.7)	1.000
Steroid	0	1 (2.3)	0.166
Epigastric pain	2 (6.7)	0	0.411
	5 (16.7)	0	0.009

Data presented as number (%) except age; GI: gastrointestinal; NSAIDs: nonsteroidal anti-inflammatory drugs.

**Table 3:** Clinical signs and laboratory results of patients presented with hematochezia categorized by sites of bleeding as upper (UGIB) or lower (LGIB) gastrointestinal bleeding.

Variables	UGIB group *N* = 30	LGIB group N = 43	P value
Systolic blood pressure, mmHg	114.3 (18.0)	132.5 (18.7)	<0.001
Diastolic blood pressure, mmHg	66.7 (12.8)	72.8 (9.9)	0.025
Pulse rate, bpm	88.7 (15.4)	86.1 (20.3)	0.558
Hematocrit, %	26.4 (6.4)	32.3 (7.3)	0.001
Platelet count, cells/mm^3^	249133.3 (93282.6)	280511.6 (91692.1)	0.159
INR, seconds	1.2 (0.5)	1.1 (0.2)	0.100
Blood urea nitrogen (BUN), mg/dL	29.0 (17.6)	19.3 (17.8)	0.024
Serum creatinine (Cr), mg/dL	1.2 (0.8)	1.4 (1.7)	0.415
BUN/Cr ratio	26.6 (13.8)	15.5 (6.9)	<0.001
Serum albumin, g/dL	3.4 (0.6)	3.6 (0.7)	0.250

Data presented as mean (standard deviation); INR: international normalized ratio; laboratory values were measured at presentation to the emergency department.

**Table 4:** Significant factors associated with bleeding from upper gastrointestinal bleeding in patients presented with hematochezia.

Variables	Adjusted odds ratio	95% confidence interval	*P* value
Systolic blood pressure	0.725 per 5 mmHg increase	0.592–0.891	0.002
Hematocrit	0.751 per 3% increase	0.582–0.970	0.029
BUN/Cr ratio	1.11 per unit increase	1.030–1.190	0.004
